# Changes of Peripapillary Region Perfusion in Patients with Chiasmal Compression Caused by Sellar Region Mass

**DOI:** 10.1155/2021/5588077

**Published:** 2021-06-14

**Authors:** Guangxin Wang, Jian Gao, Wenjuan Yu, Yang Li, Rongfeng Liao

**Affiliations:** Department of Ophthalmology, The First Affiliated Hospital of Anhui Medical University, Hefei, Anhui 230022, China

## Abstract

**Purpose:**

To evaluate the peripapillary vessel density (pVD) and the peripapillary nerve fiber layer (pRNFL) thickness in patients with chiasmal compression caused by sellar region mass using optical coherence tomography angiography (OCTA).

**Methods:**

This is an observational, cross-sectional study of 31 patients (31 eyes) with chiasmal compression caused by sellar region mass and 34 healthy controls (34 eyes). Automated perimetry and OCTA were performed. The pVD and pRNFL thickness were compared between the two groups. The impact of tumor diameter, duration of symptoms, and cavernous sinus (CS) invasion on visual dysfunction, pVD, and pRNFL thickness was also analyzed. Furthermore, we divided the patients into two subgroups according to whether there was an absolute defect in the central visual field and evaluated their pVD and pRNFL thickness, respectively.

**Results:**

Compared to the healthy control group, there was a statistically significant decrease in pVD and pRNFL thickness in patients with chiasmal compression (*p* < 0.05), especially in patients with substantial absolute defects in the central visual field. Tumor diameter, duration of symptoms, and CS invasion did not appear to be associated with pVD and pRNFL thickness. There was a significant positive correlation between the pVD and pRNFL thickness in patients with chiasmal compression (*p* < 0.001).

**Conclusion:**

pVD and pRNFL thickness are significantly decreased in patients with chiasmal compression revealed by OCTA, especially in patients with more severe visual field defects. A significant correlation between pVD and pRNFL thickness was demonstrated, which provides a clue for the study of the mechanism of changes in retinal perfusion in compressive optic neuropathy. It requires considerable attention that OCTA may play an important role in disease monitoring of sellar region mass. Hence, further studies are needed to verify whether OCTA is helpful to predict the prognosis of visual function after decompression surgery.

## 1. Introduction

The sellar region constitutes a complex structure adjacent to the optic chiasm. Sellar mass lesions include pituitary adenomas, craniopharyngiomas, Rathke's cleft cysts, and, less commonly, meningiomas, germinomas, and hamartomas [1]. Anterior visual pathway compression caused by sellar region masses is a common neuro-ophthalmological disorder. Its main symptom is the decrease of the visual acuity and visual field defects in specific areas [2–4]. Although magnetic resonance imaging (MRI) is now an essential and useful tool for managing patients with sellar tumors, there are limited objective measures shown to be predictive of postoperative visual recovery [5]. Chiasmal compression caused by sellar tumor leads to the damage and degeneration of retinal ganglion cells (RGCs) and their axons [6]; in this case, prognosis for visual recovery is often poor [7]. MRI provides structural imaging of the soft tissues from the visual system but not the microstructure. This poses a new problem, how can we observe the microstructure of retina.

Optical coherence tomography (OCT) has provided a breakthrough in observing and evaluating the microstructure of retina in vivo. In recent years, OCT has been applied clinically to monitor the disease progression and the loss of RGCs in patients with sellar tumors. Previous studies have shown that more severe preoperative atrophy of RNFL is a harbinger of poor postoperative visual function [8, 9]. Previous studies have confirmed that peripheral nerve compression can lead to changes in the perineural microcirculation which plays an important role in regulating nerve microenvironment, providing metabolic demand, and maintaining nerve conduction and axonal transport [10]. As an extension of optic nerve, will the microcirculation of retina also change?

OCTA is an advanced technology based on OCT, which enables a noninvasively visualized retinal vascular network. We were able to objectively quantify retinal microcirculation, benefiting from the reliable high-resolution images provided by OCTA. Some researchers have already used OCTA technology to illustrate the changes of retinal microcirculation and its correlation with visual dysfunction in patients with chiasmal compression [11–14]. However, the underlying mechanisms of retinal microcirculation changes remain undefined.

This study will thus quantitatively evaluate pVD and pRNFL thickness in patients with chiasmal compression caused by sellar tumor to further verify the changes using noninvasive detection OCTA and elucidate the relationship between pVD, pRNFL thickness, and visual dysfunction. Furthermore, the secondary aims were to explore the influential factors and possible mechanisms of retinal microcirculation and microstructure changes in patients with chiasmal compression.

## 2. Materials and Methods

### 2.1. Subjects

This is an observational cross-sectional, single-center study. Data were collected from 31 eyes of 31 patients with sellar tumor and 34 eyes of 34 healthy controls. Only a single eye with the worst visual field defect based on absolute defect value of the patients was selected for analysis. The patients included in this study were collected in the Department of Neurosurgery of the First Affiliated Hospital of Anhui Medical University between 2019 and 2020. This study was performed in adherence to the tenets of the Declaration of Helsinki and was approved by the institutional review board of the First Affiliated Hospital of Anhui Medical University. Informed consent was obtained from all participants.

All the patients underwent comprehensive ophthalmic examinations including best-corrected visual acuity (BCVA), which was converted to the logMAR, slit-lamp biomicroscopy, intraocular pressure (IOP), funduscopy, standard automated perimetry (Octopus 900 perimetry, Haag-Streit, Switzerland), and OCTA (Optovue, Inc., CA, USA).

The present study included patients with chiasmal compression caused by sellar region mass confirmed by MRI; all of whom have varying degrees of visual field defects in either or both eyes with or without decreased visual acuity. All patients and healthy controls have good cognitive ability and can complete all examinations in cooperation. The OCTA scanning quality index is higher than 5 and the perimetry reliability factor value less than 15. And patients who had never received any form of treatment including craniocerebral surgery, radiation, and medication were included in the study. Those with any ocular disease (including high myopia, lens opacities, uveitis, glaucoma, ptosis, a history of any retinal disease, or intraocular surgery that may affect the vessel density and visual field), diabetes, hypertension, and any other systemic diseases that may affect retinal perfusion were excluded.

### 2.2. OCTA Scanning

**Figure 1:**
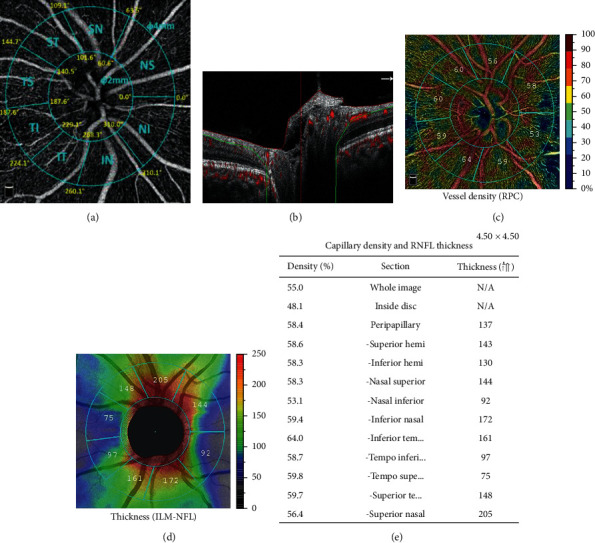
A rectangle scanning area of 4.5^*∗*^4.5 mm centered on the optic nerve head was measured. (a) The software automatically divides the round annular area, which was defined as peripapillary, into eight sectors, including nasal superior (NS), nasal inferior (NI), inferior nasal (IN), inferior tempo (IT), tempo inferior (TI), tempo superior (TS), superior tempo (ST), and superior nasal (SN). (b) The software evaluated the peripapillary vessel density (pVD) and RNFL thickness of the radial peripapillary capillaries (RPC) layer, which extends from the layer between the internal limiting membrane (ILM) to RNFL. The color map shows the vessel density (c) and the RNFL thickness (d). (e) The vessel density and RNFL thickness of each sector were quantified.

The OCTA imaging system provides a noninvasive method for visualizing the retinal vasculature and characterizes vascular information at each retinal layer as an en face angiogram, a vessel density map. Vessel density (VD) is calculated as the percentage area occupied by flowing blood vessels in the selected region. In the present study, angiography imaging of the optic disc was performed by OCTA device (Optovue, Inc., CA, USA). A 4.5 × 4.5 mm rectangle scan centered on the optic nerve head (ONH) was performed. Only well-centered images with scan quality index (SQI) > 5, without motion or blinking, were analyzed. The newly developed, built-in Angio Analytics software (version 2017.1.0.151; Optovue, Inc.) was applied to evaluate pVD and pRNFL thickness of the radial peripapillary capillaries (RPC) layer, which is defined as the layer between the internal limiting membrane (ILM) to RNFL. Parameters were reported in eight sectors segmented automatically (nasal superior, nasal inferior, inferior nasal, inferior tempo, tempo inferior, tempo superior, superior tempo, and superior nasal). The average pVD was also measured (Figure 1).

### 2.3. Visual Field Testing

All patients received the full visual field test, including central 30° and peripheral (30°–70°) using the automated perimeter (Octopus 900, Haag-Streit Inc., Koenic, Switzerland) with the Semiquantitative 07 Standard program. Depending on refraction and age, near corrective lenses were provided for testing. The background luminance of the cupola of the Octopus 900 perimeter was automatically adjusted to 31.4 asb, and the maximum stimulus luminance was 4000 asb. The intensity of that initial stimulus was 4 dB below the normal sensitivity threshold for each test point. The test point is recorded as normal if the patient can see it. If the patient cannot see it, adjust the threshold of the second stimulus to 0 dB, which is the maximum stimulus luminance. The position is marked as a relative defect if the patient can see the optotype at this time. If the patient still cannot see it, the test point is marked as an absolute defect. Stimulus appears randomly, and the time of each occurrence is 100 ms. A total of 130 test points were recorded. The visual function of each test point is divided into three levels, including normal, relative defect, and absolute defect, as described above. A reliability factor value less than 15 is considered to be reliable. Patients were divided into two subgroups according to whether there is an absolute defect in the central visual field.

### 2.4. Statistical Analysis

SPSS software (version 20.0) for Windows was applied to statistical analysis. The Shapiro-Wilk test was used to study the normality of the data. All data are shown as the mean ± standard deviation (SD), median, and interquartile range (IQR, 25th–75th). The data were compared between the patients and the healthy controls by Mann-Whitney *U* test for nonnormal variables, unpaired *t*-test for normally distributed variables, and analysis of covariance to adjusting the age. The area under the receiver operating characteristic curves (AUC) were used to describe the ability of OCTA vessel densities to discriminate eyes with chiasmal compression from control eyes. Respective correlation between the pVD and pRNFL was examined by using the Spearman rank correlation and Pearson Correlation Coefficient. Use multiple linear regression to analyze the influence of tumor diameter and symptom duration on the BCVA, pVD, and pRNFL thickness. A *p* value < 0.05 was considered statistically significant.

## 3. Results

### 3.1. Demographics and Clinical Characteristics of the Patients

A total of 31 eyes of patients with chiasmal compression caused by sellar tumor and 34 healthy control eyes were involved in the present study. Demographic and clinical characteristics of participants are shown in Table 1. No statistical differences were found in age and gender between the patients and the healthy control group overall (*p* > 0.05). Age showed statistical differences in patients without central visual field defects. Compared with the control group, after adjusting the age, the BCVA value of the patients decreased significantly (*p* < 0.05). There was no statistical significance between the two subgroups. 68% of patients had BCVA of 6/12 or better. 45% of patients had BCVA of 6/7.5 or better, and 29% of them had BCVA of 6/6 or greater. Patients consisted of pituitary adenomas (26, 83.9%), craniopharyngiomas (3, 9.7%), meningioma (1, 3.2%), and metastases (1, 3.2%). The duration of patients' clinical symptoms ranged from 0.2 to 36 months. Among them, 14 patients (45.2%) sought medical treatment because of visual impairment. 11 patients had cavernous sinus (CS) invasion on the side with severe visual field defect. No obvious CS invasion was found in the other 20 patients.

### 3.2. Comparison of pVD and pRNFL Thickness between the Patients and Healthy Controls

**Figure 2:**
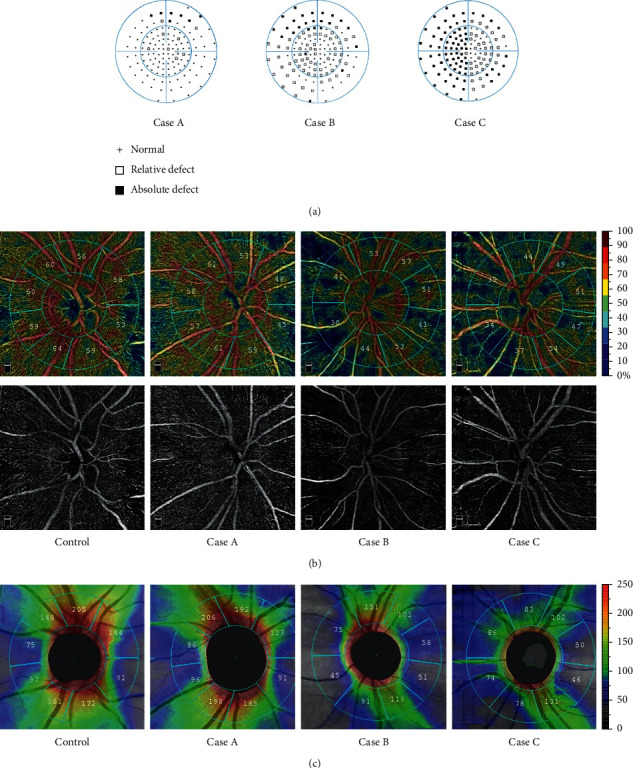
Samples from a healthy control subject and three cases with visual field defect of different degrees. (a) Visual field measured by Semiquantitative 07 Standard program. There was an absolute defect in the central visual field in case C, while A and B mainly involved the peripheral visual field. The color map and en face OCTA of vessel density (a) and RNFL thickness (b) of each sample are shown in the figure. The color maps suggest that the pVD and pRNFL thickness of patients with more severe visual field defects are significantly decreased.

**Figure 3:**
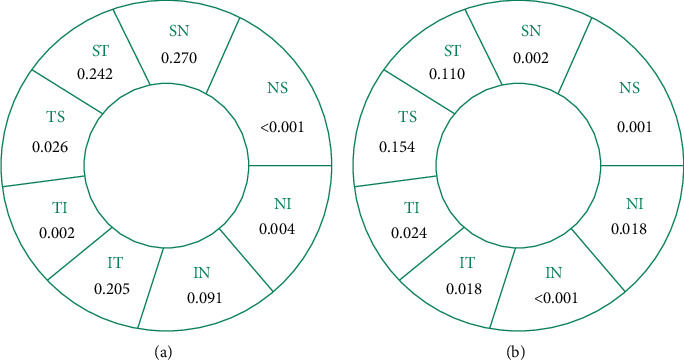
*p* value of the comparison of pVD (a) and pRNFL thickness (b) in eight sectors between patients with chiasmal compression caused by sellar tumor and healthy controls. Significant decreases in pVD were observed in the NS, NI, TI, and TS sectors (*p* < 0.05). Statistically significant thinning of the RNFL thickness was observed except for the TS and ST sectors.

The color map and en face OCTA of pVD for one representative sample from a healthy control subject and three cases with chiasmal compression were shown in Figure 2(b).  Table 2 shows the comparison of pVD between both groups. There were statistically significant decreases in the pVD in the peripapillary annulus (*p* < 0.05). Among the eight sectors of the peripapillary annulus, significant decreases in pVD were observed in the nasal superior, nasal inferior, tempo inferior, and tempo superior (*p* < 0.05), as shown in Figure 3(a). The highest diagnostic ability was the pVD in nasal superior sectors (AUC = 0.763), followed by the tempo inferior (AUC = 0.709). Figure 2(c) shows the color map of pRNFL thickness for the samples. The RNFL thickness value for each group was presented in Table 2. The average pRNFL thickness was significantly thinner than that of the control group (*p* < 0.05). In the divided eight sectors, statistically significant thinning of the pRNFL thickness was observed in all but tempo superior and superior tempo sectors (Figure 3(b)). The best performing sector was the inferior nasal (AUC = 0.787). Correlation between pVD and pRNFL thickness in patients is reported in Table 3. There was a significant positive correlation between pVD and pRNFL thickness (*p* < 0.001), mainly in nasotemporal and superior nasal sectors.

### 3.3. Comparison of pVD and pRNFL Thickness between Patients with and without Central Visual Field Involvement and Healthy Controls

We divided the patients into two subgroups according to whether the absolute defect of the visual field involved the central 30° (Table 4). pVD and pRNFL thickness decreased significantly in patients with central visual field defects than those in healthy controls (*p* < 0.001) and patients without significant central visual field involvement (*p* < 0.05). Compared to the healthy control group, although the pVD and pRNFL thickness of patients with no absolute central visual field defect decreased, it did not reach statistical significance (*p* > 0.05). Three cases with different degrees of visual field deficit are shown in Figure 2. There was an absolute defect in the central visual field in case C, while cases A and B mainly involved the peripheral visual field. A qualitative comparison of pVD, pRNFL thickness, and visual field revealed that decreases in pVD and pRNFL thickness are more significant in patients with more severe visual field defects.

### 3.4. The Effects of Tumor Diameter and Symptom Duration on Visual Impairment, pVD, and pRNFL Thickness

We did not find that tumor diameter and duration of symptoms were related to pVD and pRNFL thickness, whether it is transverse diameter, vertical diameter, or anteroposterior diameter (shown in Table 5). However, the vertical diameter of the tumor seems to have a negative effect on BCVA. The vertical diameter of the tumor in patients with absolute defects in the central visual field is significantly larger than that in patients without absolute defects in the central visual field after adjusting the age (*F* = 4.230, *p*=0.049), while there are no significant differences in the symptom duration (*F* = 0.778, *p*=0.385), transverse (*F* = 2.215, *p*=0.148), and anteroposterior (*F* = 1.315, *p*=0.261) diameters of the tumor.

### 3.5. Effect of CS Invasion on pVD and pRNLF Thickness

We analyzed whether CS invasion would affect pVD and pRNFL thickness (Table 6). We found that the CS invasion appeared to have no significant effect on pVD and pRNFL thickness (*p* > 0.05).

## 4. Discussion

Previous studies have evaluated the changes of retinal structure, such as the thicknesses of RNFL and the ganglion cell complex (GCC) in patients with sellar region mass based on OCT [6, 15, 16]. However, few studies reported the changes of retinal microvasculature in chiasmal compression. Fundus fluorescein angiography (FFA) is regarded as the gold standard for diagnosing retinal and choroidal vascular abnormalities [17]. However, it has several limitations, such as allergy caused by the injection of fluorescent dye, an inability to image the RPC networks well, and an inability to quantify ischemia degrees. OCTA, the latest noninvasive imaging modality that uses motion contrast to generate angiography images, was applied to this study. OCTA provides reliable, high-resolution, and noninvasive images of the retinal vasculature efficiently. These images are approaching histology level resolution [18] and have high repeatability [19].

In our study, we evaluated the VD and RNFL thickness changes in the peripapillary region of patients with chiasmal compression caused by sellar tumors. A statistically significant decrease in VD of RPC segment was found in the peripapillary region compared to the healthy controls, especially in the nasal and temporal quadrants (Figure 3(a)). We also found that, compared with the healthy control group, the thickness of the pRNFL in patients with chiasmal compression was significantly thinner, especially in the nasal and inferior sectors (Figure 3(b)), which was similar to the results of the recent publication by Ga-In Lee et al. [12] and Laura Dallorto et al. [11]. Additionally, we also found a statistically significant positive correlation between pVD and pRNFL thickness in patients with chiasmal compression.

At present, it is considered that the mechanisms of chiasmal compression affecting visual function include axoplasmic stasis, conduction block, demyelination, and axonal damage. Retinal ganglion cell loss and axonal damage were regarded as having an effect on the measured thickness of RNFL [2, 7]. The RPC originates from the peripapillary retinal arterioles around the optic disc and extends radially from the optic disc, parallel to the RNFL axon and lying among the superficial nerve fibers, providing blood and nutrition for RGCs [20]. Previous studies have found that there is a positive correlation between the RPC volume and the RNFL thickness in the normal human retina, which indicates a necessary supportive role of the RPC in the RNFL [21]. The correlation we found between pVD and pRNFL thickness in patients with chiasmal compression seemed to be attributed to the decrease of nutrient demand due to loss of ganglion cells and axonal injury, which leads to a secondary decrease in regional perfusion [11, 13].

Mitochondrial-rich varicosities suggest high energy demands for nonmyelinated axons in RNFL. The high energy requirements of axons make them very vulnerable to injury, especially from ischemic insults [21, 22]. Numerous studies also have demonstrated a significant correlation between RPC and RNFL thickness in a variety of diseases, such as diabetic retinopathy, retinal vein occlusion, and ischemic optic neuropathy [21, 23–25]. The study of the correlation is helpful to improve our understanding of the pathogenic relationship between the microcirculation around the optic disc and the process of axon loss. Among these diseases, the decrease in peripapillary perfusion is thought to be possibly related to primary vasculature dysfunction and is involved in the process of axonal damage.

The anatomical structure around the optic chiasm is complex, with the internal carotid arteries adjacent to it and located on both sides, possibly affected by a nearby space-occupying lesion [26, 27]. Blood flow to the retina is mainly provided by the internal carotid artery, which therefore plays an important role in ocular microcirculation [28]. It is widely known that the CS contains the carotid artery and some of its branches [29]. To verify whether there is a vascular mechanism in pVD reduction and axonal injury, we compared the pVD and pRNFL thickness in patients with CS invasion and patients without CS invasion. No significant differences were found. Therefore, changes in RPC are more likely to occur secondary to the loss of ganglion cells and axonal injury. Our findings may provide useful information. With the development of OCTA technology, further cross-sectional and longitudinal studies are expected to clarify the potential mechanism.

Tumor size is considered as one of the risk factors for preoperative visual dysfunction. Previous studies have shown that there is a close correlation between the tumor diameter and the visual dysfunction [30], but some studies believe that there is no close correlation between them [31]. Therefore, we analyzed the effect of tumor diameter on visual impairment, pVD, and pRNFL thickness. The vertical diameter of the tumor was related to the visual acuity and visual field defect, while others were not significantly associated with visual function, pVD, and pRNFL thickness. The position of the optic chiasm relative to the tumor which has large individual differences is thought to be more important in determining the visual impairment. The direction of the compressive force and the deformation pattern of the optic chiasm are important determinants of the degree of visual dysfunction [32].

Some researchers have suggested that there is a correlation between the duration of symptoms and visual impairment [33], while other researchers reported no correlation between the duration of symptoms and the visual dysfunction [34]. Our study also verified that the duration of symptoms is not related to visual dysfunction, pVD, and pRNFL thickness. Therefore, the duration of symptoms may not be an important impact factor in the visual system damage of patients with optic chiasm compression. For example, patients with pituitary apoplexy often have a very severe visual impairment, even if the duration of symptoms may be as short as 24 hours [2].

We observed a statistically significant decrease in pVD and pRNFL thickness in patients with absolute defects involving central visual field compared with healthy controls and patients without central visual field involvement. However, a statistically significant decrease was not observed in patients without central visual field defects. A descriptive study of retinal microstructures by Blanch in seven patients without a significant visual field defect in central 30° shows that the RNFL thickness was thinner than normal in 5 patients [7]. Although there was no statistically significant decrease in our study, we found observable changes in microvasculature and microstructures compared to healthy control in some patients with absolute defects only in the peripheral visual field (Figure 2). The lack of detection of a statistically significant decrease may be related to the small sample size or the varying degree of disease progression, which can also be explained by the limited scan range of the OCTA since Higashiyama demonstrated that the decreased peripapillary retinal perfusion was associated with quadrants of visual field defects caused by chiasmal compression [14].

Among the patients included in the study, 68% of patients had BCVA of 6/12 or better, 45% of patients had BCVA of 6/7.5 or better, and 29% of them had BCVA of 6/6 or greater. This is consistent with the results of previous studies. More than half of patients have visual field defects but still retain good visual acuity [35]. We found that, in previous studies, sellar tumor patients all received visual field examination to evaluate the central 30° of the visual field [11–13, 36]. However, visual field defects may first appear on the periphery and affect the central visual field and visual acuity in the later stage of progression [37]. In this case, examining the central field only in patients with sellar tumors shall not be adequate, and the peripheral field is also essential. Therefore, in the diagnosis and treatment of patients with sellar mass, a full visual field examination program and objective indicators are needed.

This study has several limitations. First of all, the sample size we included was small. A study with a larger sample size is required to validate our findings and allow us to evaluate the different compression areas and different morphological distortions of the optic chiasm caused by sellar tumors and their effects on visual impairment, retinal microstructures, and perfusion. Second, with a 4.5 × 4.5 mm rectangle scan centered on the ONH performing, we only obtained the quantitative data of the posterior pole; thus, there was a lack of observation of the peripheral retina. Finally, there was a lack of longitudinal comparison to demonstrate the predictive value of pVD and pRNFL thickness for visual function and retinal structural recovery after decompression surgery. A follow-up study with a larger sample size is ongoing.

In conclusion, marked reductions in pVD and pRNFL thickness were observed in eyes with chiasmal compression compared with healthy controls in our study. This change is more pronounced in patients with more severe visual field defects involving the central visual field. Moreover, there is a significant correlation between pVD and pRNFL thickness in patients with chiasmal compression. The application of OCTA in patients with sellar tumors may help us understand the various retinal pathological changes in patients and the mechanism of retinal perfusion changes in compressive optic neuropathy. Besides, the visual field examination is subjective and requires high cooperation and concentration. pVD and pRNFL thickness may prove to be the biomarker capable of evaluating the disease severity and progression as an objective measure, especially among patients with limited cooperation. Further longitudinal studies are needed to determine the visual prognostic value of OCTA.

## Figures and Tables

**Table 1:** Demographics and clinical characteristics of patients with and without absolute central defect and the healthy control.

	Sellar tumor overall	Group 1	Group 2	Group 3	*p* value	*p* value	*p* value	*p* value
					ST versus 3	1 versus 3	2 versus 3	1 versus 2
Patients (*n*)	31	19	12	34	—	—	—	—
Eyes (*n*)	31	19	12	34	—	—	—	—
Sex								
Female (*n*)	17	11	6	13	—	—	—	—
Male (*n*)	14	8	6	21	0.180	0.168	0.477	0.667
Age (years)	48.94 ± 14.31	52.95 ± 15.12	42.58 ± 10.63	50.15 ± 8.24	0.682	0.462	**0.015**	**0.048**
BCVA; logMAR	0.36 ± 0.48	0.43 ± 0.44	0.26 ± 0.56	0.04 ± 0.05	**0.001**	**<0.001**	**0.038**	0.306

Group 1, patients with an absolute defect in the central visual field. Group 2, patients without an absolute defect in the central visual field. Group 3, healthy controls. ST = sellar tumor. BCVA = best-corrected visual acuity. Bold values are statistically significant values (*p* < 0.05).

**Table 2:** Comparison of pVD and pRNFL thickness between the patients and the healthy controls.

	Sellar tumor	Healthy control	*z*/*t* value	*p* value	AUC (*p*)
pVD (%)	49.34 ± 6.64	53.31 ± 2.21	−3.174	**0.003**	**0.695 (0.007)**
Nasal superior	46.20 (41.70–49.50)	49.60 (48.55–53.15)	−3.646	**< 0.001**	**0.763 (< 0.001)**
Nasal inferior	44.80(37.50–49.80)	49.40 (45.83–51.65)	−2.844	**0.004**	**0.705 (0.004)**
Inferior nasal	51.80 (48.10–55.30)	53.75 (51.23–56.10)	−1.688	0.091	0.622 (0.091)
Inferior tempo	58.20 (54.70–61.50)	59.70 (57.08–61.83)	−1.268	0.205	0.592 (0.205)
Tempo inferior	48.66 ± 8.11	53.92 ± 3.98	−3.267	**0.002**	**0.709 (0.004)**
Tempo superior	54.20 (49.00–58.00)	57.70 (53.58–60.13)	−2.227	**0.026**	**0.661 (0.026)**
Superior tempo	54.50 (49.20–60.20)	56.15 (53.30–58.75)	−1.169	0.242	0.584 (0.242)
Superior nasal	51.50 (46.90–53.70)	51.95 (48.28–55.08)	−1.104	0.270	0.580 (0.270)

pRNFL thickness (*μ*m)	103.45 ± 20.69	118.41 ± 9.48	−3.689	**0.001**	**0.766 (< 0.001)**
Nasal superior	96.03 ± 23.35	112.94 ± 15.73	−3.391	**0.001**	**0.724 (0.002)**
Nasal inferior	80.00 (64.00–93.00)	91.50 (83.00–99.50)	−2.372	**0.018**	**0.671 (0.018)**
Inferior nasal	129.06 ± 26.21	154.91 ± 24.13	−4.140	**< 0.001**	**0.787 (< 0.001)**
Inferior tempo	141.71 ± 29.87	156.53 ± 16.30	−2.450	**0.018**	**0.666 (0.022)**
Tempo inferior	67.61 ± 19.82	77.50 ± 13.43	−2.332	**0.024**	**0.668 (0.020)**
Tempo superior	73.00 (58.00–93.00)	81.50 (75.00–88.25)	−1.426	0.154	0.603 (0.154)
Superior tempo	125.00 (112.00–146.00)	141.50 (123.75–149.00)	−1.596	0.110	0.615 (0.111)
Superior nasal	121.32 ± 33.19	144.38 ± 25.21	−3.171	**0.002**	**0.731 (0.001)**

pVD = peripapillary vessel density. pRNFL thickness = peripapillary retinal nerve fiber layer thickness. AUC = area under the receiver operating characteristic curves. Bold values are statistically significant values (*p* < 0.05).

**Table 3:** Correlations between pVD and pRNFL thickness in corresponding sectors of patients.

VD (%)	RNFL thickness (*μ*m)	
	*r* value	*p* value
Peripapillary	0.779	**<0.001**
Nasal superior	0.674	**<0.001**
Nasal inferior	0.608	**<0.001**
Inferior nasal	0.295	0.107
Inferior tempo	0.267	0.146
Tempo inferior	0.647	**<0.001**
Tempo superior	0.811	**<0.001**
Superior tempo	0.229	0.215
Superior nasal	0.630	**<0.001**

VD = vessel density. RNFL thickness = retinal nerve fiber layer thickness. Bold values are statistically significant values (*p* < 0.05).

**Table 4:** Comparison of pVD and pRNFL thickness between patients with or without absolute central visual deficit and healthy controls.

	Group 1	Group 2	Group 3	*p* value (1 versus 2)	*p* value (1 versus 3)	*p* value (2 versus 3)
pVD (%)	47.39 ± 7.48	52.42 ± 3.42	53.31 ± 2.21	**0.027**	**<0.001**	0.273
pRNFL thickness (*μ*m)	96.63 ± 18.61	114.25 ± 19.82	118.41 ± 9.48	**0.015**	**<0.001**	0.178

Group 1, patients with an absolute defect in the central visual field. Group 2, patients without an absolute defect in the central visual field. Group 3, healthy controls. pVD = peripapillary vessel density. pRNFL = peripapillary retinal nerve fiber layer. Bold values are statistically significant values (*p* < 0.05).

**Table 5:** Multivariate linear regression analysis of the effects of tumor diameter and symptom duration on BCVA, pVD, and pRNFL thickness.

	BCVA		pVD		pRNFL thickness	
	*β*	*p*	*β*	*p*	*β*	*p*
Transverse diameter	−0.050	0.058	−0.074	0.849	0.947	0.418
Anteroposterior diameter	−0.021	0.366	−0.139	0.686	−0.781	0.449
Vertical diameter	0.051	**0.029**	−0.098	0.772	−0.876	0.390
Symptom duration	−0.001	0.935	0.065	0.618	0.498	0.208

BCVA = best-corrected visual acuity. pVD = peripapillary vessel density. pRNFL = peripapillary retinal nerve fiber layer. Bold values are statistically significant values (*p* < 0.05).

**Table 6:** Comparison of pVD and pRNLF thickness in patients with and without cavernous sinus invasion.

	CS invasion	No CS invasion	*F* value	*p* value
pVD	49.83 ± 5.16	49.07 ± 7.44	0.030	0.863
pRNFL	102.00 ± 16.92	104.25 ± 22.87	0.208	0.652

CS = cavernous sinus. pVD = peripapillary vessel density. pRNFL = peripapillary retinal nerve fiber layer.

## Data Availability

The primary data used to support the finding of this study can be obtained upon request to the first author through e-mail (wgx725@163.com).
